# Comparison of the quality of ovarian tissue cryopreservation by conventional slow cryopreservation and vitrification—a systematic review and meta-analysis

**DOI:** 10.1186/s13048-024-01561-7

**Published:** 2025-03-26

**Authors:** Qingduo Kong, Cheng Pei, Gohar Rahimi, Peter Mallmann, Volodimir Isachenko

**Affiliations:** 1 Department of Obstetrics and Gynecology, Medical Faculty, Cologne University, 50931 Cologne, Germany; 2 Medizinisches Versorgungszentrum AMEDES für IVF- und Pränatalmedizin in Köln GmbH, 50968 Cologne, Germany

**Keywords:** Cryopreservation, Vitrification, Ovary or ovarian tissue, Ovarian follicles

## Abstract

**Background:**

Ovarian tissue cryopreservation is increasingly applied in patients undergoing gonadotoxic radiotherapy or chemotherapy treatment or other patients who need to preserve their fertility. However, there is currently limited evidence to know which type of ovarian tissue cryopreservation is better. The advantages and disadvantages of conventional slow cryopreservation and vitrification are still controversial. The purpose of this meta-analysis was to analyze the ovarian tissue quality of ovarian tissue cryopreservation by conventional slow cryopreservation and vitrification.

**Methods:**

According to the keywords, Pubmed, Embase, and Cochrane Library were searched for studies to January 2024. Studies comparing the follicular viability of conventional slow cryopreservation versus vitrification were assessed for eligibility. The meta-analysis was performed using Stata software (Version 12.0) and Review Manager (Version 5.2).

**Results:**

A total of 18 studies were included in this meta-analysis. The pooled results of the primary outcomes indicated that there was no difference between the two approaches for follicular viability (RR = 0.96, 95% CI: 0.84–1.09, *P* = 0.520, I^2^ = 95.8%, Random-effect), the proportion of intact primordial follicles (RR = 1.01, 95% CI: 0.94–1.09, *P* = 0.778, I^2^ = 70.6%, Random-effect). The pooled results of the secondary outcomes indicated that there was no difference between the two approaches for the proportion of DNA fragmented follicles (RR = 1.20, 95% CI: 0.94–1.54, *P* = 0.151, I^2^ = 0.0%, Fixed-effect), and the proportion of stromal cells (RR = 0.58, 95% CI: 0.20–1.65, *P* = 0.303, I^2^ = 99.7%, Random-effect).

**Conclusions:**

Conventional slow cryopreservation and vitrification appear to provide comparable outcomes. The heterogeneity of the literature prevents us from comparing these two techniques. Further high-quality studies are needed to enhance this statement. This meta-analysis provides limited data which may help clinicians when counselling patients.

## Introduction

Ovarian tissue cryopreservation has become a widely adopted and significant method for fertility preservation as an increasing number of women become aware of its benefits [1–4]. Compared with oocyte cryopreservation, ovarian tissue cryopreservation is the only option for preserving fertility in women undergoing cancer treatment that cannot be delayed or in prepubertal girls for whom mature germ cells are not available [5–10]. Additionally, ovarian cryopreservation can also be applied to patients with ovarian benign tumors that require ovarian removal and premature ovarian failure. Advancements in autologous transplantation technology following ovarian cryopreservation are also making significant strides, enabling the restoration of endocrine function [11–15].

Since the birth of the first baby using ovarian tissue cryopreservation in 2004, more than 200 babies have been born worldwide through this technology [16, 17]. There are currently two main cryopreservation methods, respectively conventional slow freezing and vitrification [18, 19]. Before cryopreservation, the ovarian tissue should be obtained by laparoscopy or laparotomy. The ovarian tissue with immature germ cells of one or both ovaries is separated into small pieces about 5 × 5 mm^2^ with a thickness of 1–3 mm before cryopreservation [20].

Conventional slow freezing uses a controlled-rate freezer to gradually cool ovarian tissue with a lower concentration of cryoprotectant, minimizing ice crystal formation and reducing cell damage. Cryoprotectants help limit intracellular ice but may be cytotoxic. Extending freezing time and lowering cryoprotectant levels further reduce cell damage risks. After freezing, the tissue is stored in cryovials within liquid nitrogen at around − 196 °C [21, 22].

Vitrification rapidly cools ovarian tissue to minimize intracellular ice formation, using higher concentrations of cryoprotectants like Dimethyl sulfoxide (DMSO), glycerol, propylene glycol (PrOH), or ethylene glycol (EG) as osmotic agents. While high concentrations increase cytotoxicity, they reduce tissue stress by limiting ice formation. Minimizing exposure to these agents also lowers cell damage risk. Rapid cooling turns the solution into a glass-like, amorphous state, and the tissue is stored in liquid nitrogen at approximately − 196 °C [23–25].

Thawing is a crucial step in cryopreservation, warming frozen tissue back to physiological temperature before transplantation. The ovarian tissue is gradually warmed to prevent intracellular ice crystal formation, using specialized warming devices. Afterward, cryoprotectants are removed, and histological examination assesses tissue quality [26].

**Fig. 1:**
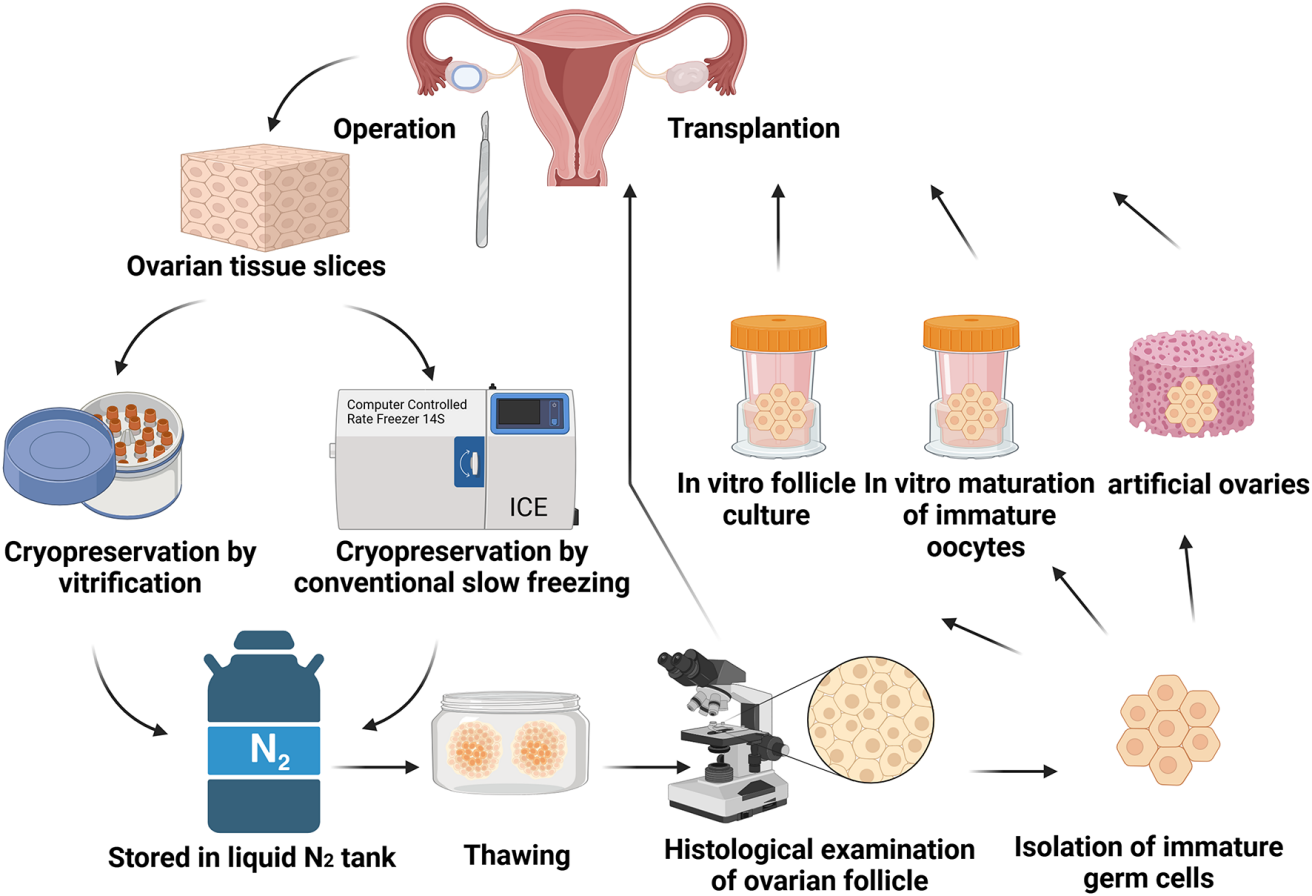
Schematic overview of ovarian cryopreservation

Histological examination of ovarian tissue is essential for evaluating the success of ovarian tissue cryopreservation and forms the basis for ovarian transplantation. Follicle viability is the most important aspect in the histological examination of ovarian tissue, serving as a key indicator of fertility [27, 28]. Common methods are hematoxylin-eosin (HE) staining, dead and alive cell detection, etc. Since freezing can increase DNA damage, assessing DNA integrity is also essential and is a critical criterion for evaluating the impact of ovarian tissue cryopreservation [29, 30]. The stromal cells of the ovary are a key component of the ovarian tissue, playing a crucial role in supporting the development and function of ovarian follicles [31]. Therefore, the viability and number of stromal cells in the ovarian tissue cryopreservation also determine the growth of follicles and ovarian function after thawing [32]. After thawing the ovarian tissue, ovarian transplantation is the best way to preserve the patient’s fertility and endocrine function. In addition, there are several other ways to culture immature follicles and then transplant them. The culture methods are in vitro follicle culture, in vitro maturation of immature oocytes, and artificial ovary [33, 34]. The schematic overview of ovarian cryopreservation is shown in Fig. 1.

This article aimed to perform a systematic review and meta-analysis to assess the condition of primary and secondary oocytes, stromal cells, and DNA damage in ovarian tissues following conventional slow freezing and vitrification, providing a quality evaluation.

## Methods

### Search strategy

The study was conducted according to the meta-analysis of observational studies in epidemiology (MOOSE) guidelines. We performed a literature search using the keywords “ovarian tissue”, “ovary”, follicle”, “cryopreservation”, and “vitrification” in various combinations to January 2024 in PubMed, Embase, and Cochrane Library. We conducted a Pubmed search using the following search string: (“ovary“[MeSH Terms] OR “ovarial“[All Fields] OR “ovary“[All Fields] OR “ovaries“[All Fields] OR “ovary s“[All Fields] OR ((“ovarian“[All Fields] OR “ovarians“[All Fields]) AND (“tissue s“[All Fields] OR “tissues“[MeSH Terms] OR “tissues“[All Fields] OR “tissue“[All Fields])) OR (“follicle s“[All Fields] OR “hair follicle“[MeSH Terms] OR (“hair“[All Fields] AND “follicle“[All Fields]) OR “hair follicle“[All Fields] OR “follicles“[All Fields] OR “ovarian follicle“[MeSH Terms] OR (“ovarian“[All Fields] AND “follicle“[All Fields]) OR “ovarian follicle“[All Fields] OR “follicle“[All Fields])) AND (“cryopreservability“[All Fields] OR “cryopreservable“[All Fields] OR “cryopreservant“[All Fields] OR “cryopreservants“[All Fields] OR “cryopreservated“[All Fields] OR “cryopreservation“[MeSH Terms] OR “cryopreservation“[All Fields] OR “cryopreserved“[All Fields] OR “cryopreservations“[All Fields] OR “cryopreservative“[All Fields] OR “cryopreservatives“[All Fields] OR “cryopreserve“[All Fields] OR “cryopreserving“[All Fields]) AND (“vitrificated“[All Fields] OR “vitrification“[MeSH Terms] OR “vitrification“[All Fields]). We conducted an Embase search using the following search string: ((‘ovary’/exp OR ovary OR ovarian) AND (‘tissue’/exp OR tissue) OR ‘follicle’/exp OR follicle) AND (‘cryopreservation’/exp OR cryopreservation) AND (‘vitrification’/exp OR vitrification). We conducted a Cochrane Library search using the following search string: (ovary OR ovarian tissue OR follicle) AND cryopreservation AND vitrification. The search All studies were assessed by two investigators independently and any difference was settled by discussion. Studies in all languages were included.

### Study selection criteria and exclusion criteria

Research articles comparing the follicular viability of conventional slow cryopreservation and vitrification were considered appropriate for the analysis. Studies that were not classified as research articles were excluded. And studies focused on other topics or with insufficient data for follicular viability were also excluded.

### Outcome measures

#### Primary outcomes

- Follicular viability (the proportion of viable follicles in the total follicles).
- The proportion of intact primordial follicles (the proportion of intact primordial follicles in the total primordial follicles).

#### Secondary outcomes

- The proportion of DNA fragmented follicles (the proportion of DNA damage follicles in the total follicles).
- The proportion of stromal cells (the proportion of intact stromal cells in the total stromal cells).

### Data abstraction and quality assessment

The following data were extracted: baseline characteristics including age range, mean age, how patients were found, the reason for exclusion of participants, surgical techniques, the method of follicles quality evaluation, the freezing solution of conventional slow freezing and vitrification, follicular density, follicular viability, the proportion of intact primordial follicles, the proportion of DNA fragmented follicles. The risk of bias in individual studies was assessed by the Funnel plot bias. The Newcastle-Ottawa Scale for evidence-based medicine checklist about each risk of bias item as percentages across all included studies were used to evaluate the methodological quality.

### Statistical analysis

This meta-analysis was performed using Stata software (Version 12.0) and Review Manager (Version 5.2). We used the Cochran Q test to evaluate the heterogeneity. Heterogeneity was used to evaluate the percentage of the variation in all studies. Because of inevitable clinical and methodologic diversity, inconsistency (I^2^) was adopted to quantify the effect of statistical heterogeneity. A value of 0% indicated no observed heterogeneity and a value of more than 50% was considered substantial heterogeneity. If I^2^ > 50%, the random-effects model was adopted, otherwise, the fixed-effects model was used to pool the results. Review Manager (Version 5.2) was used to do the risk of bias graph. A two-sided *P* < 0.05 was considered statistically significant.

## Results

### Study characteristics

**Fig. 2:**
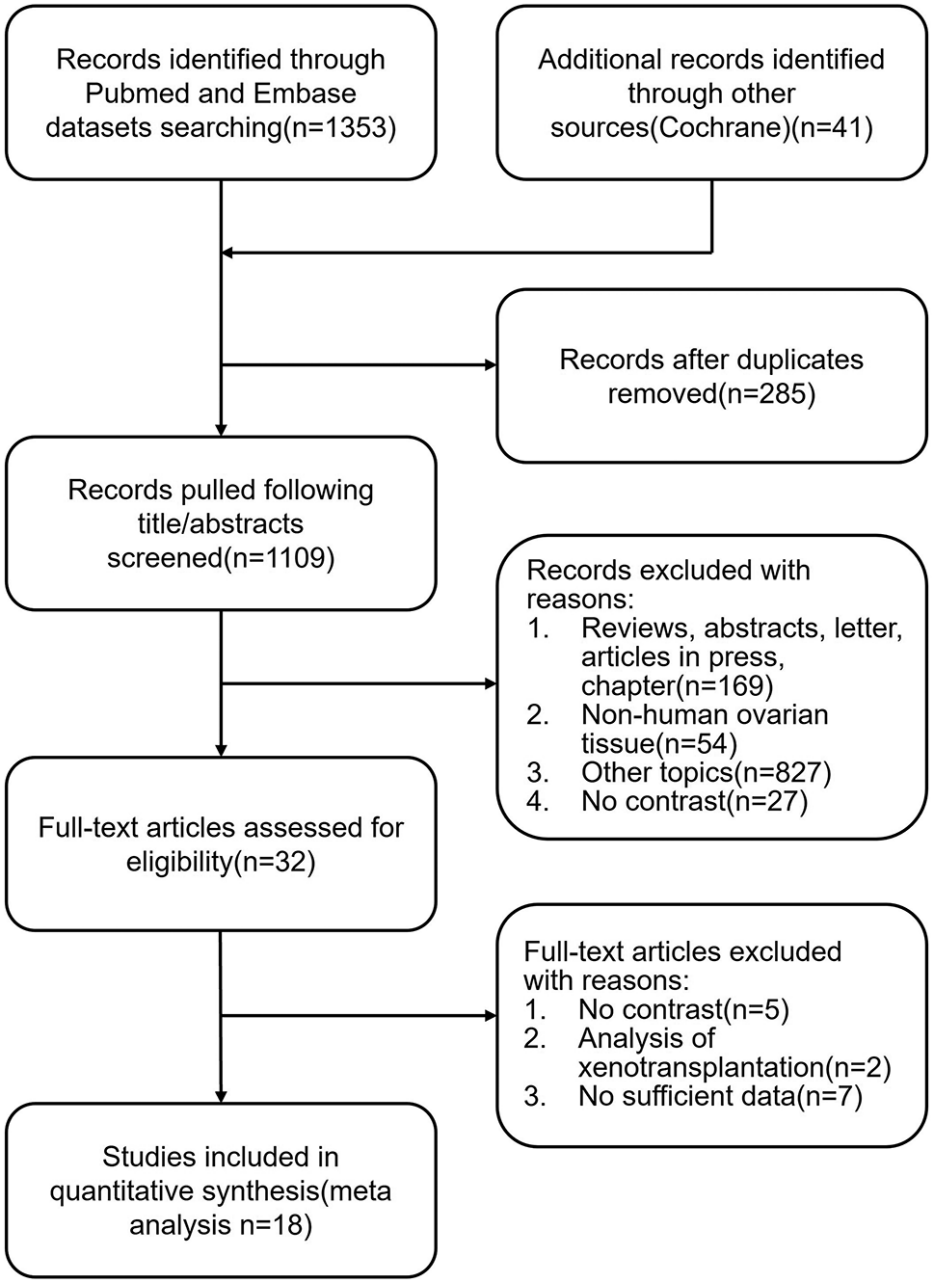
Flow diagram describing the research selection progress

The search process is shown in Fig. 2. The number of records identified through PubMed, Embase is 1353. The total number of records is 1394 added the records identified through the Cochrane Library. After screening titles and abstracts, 32 publications remained. In the 32 publications, 5 of them have no comparison group, 2 of them are the analysis of xenotransplantation, and 7 of them have no sufficient data. Finally, 18 publications were selected for this meta-analysis [35–52].

Details of the 18 publications are shown in Tables 1 and 2. All the included studies are published between 2007 and 2022. Published at most in 2022 is 3 articles. The included studies are from 10 countries, China for 5 studies is the most, and Germany for 3 is the second. The mean age range is nearly 20–40 years old. Laparoscopy surgery is the most common surgical approach 7 publications use laparoscopy surgery to obtain ovarian tissue, and 4 publications use laparoscopy surgery or open surgery to obtain ovarian tissue. All cryopreservation ovarian tissue was analyzed by light microscopy.

**Table 1:**
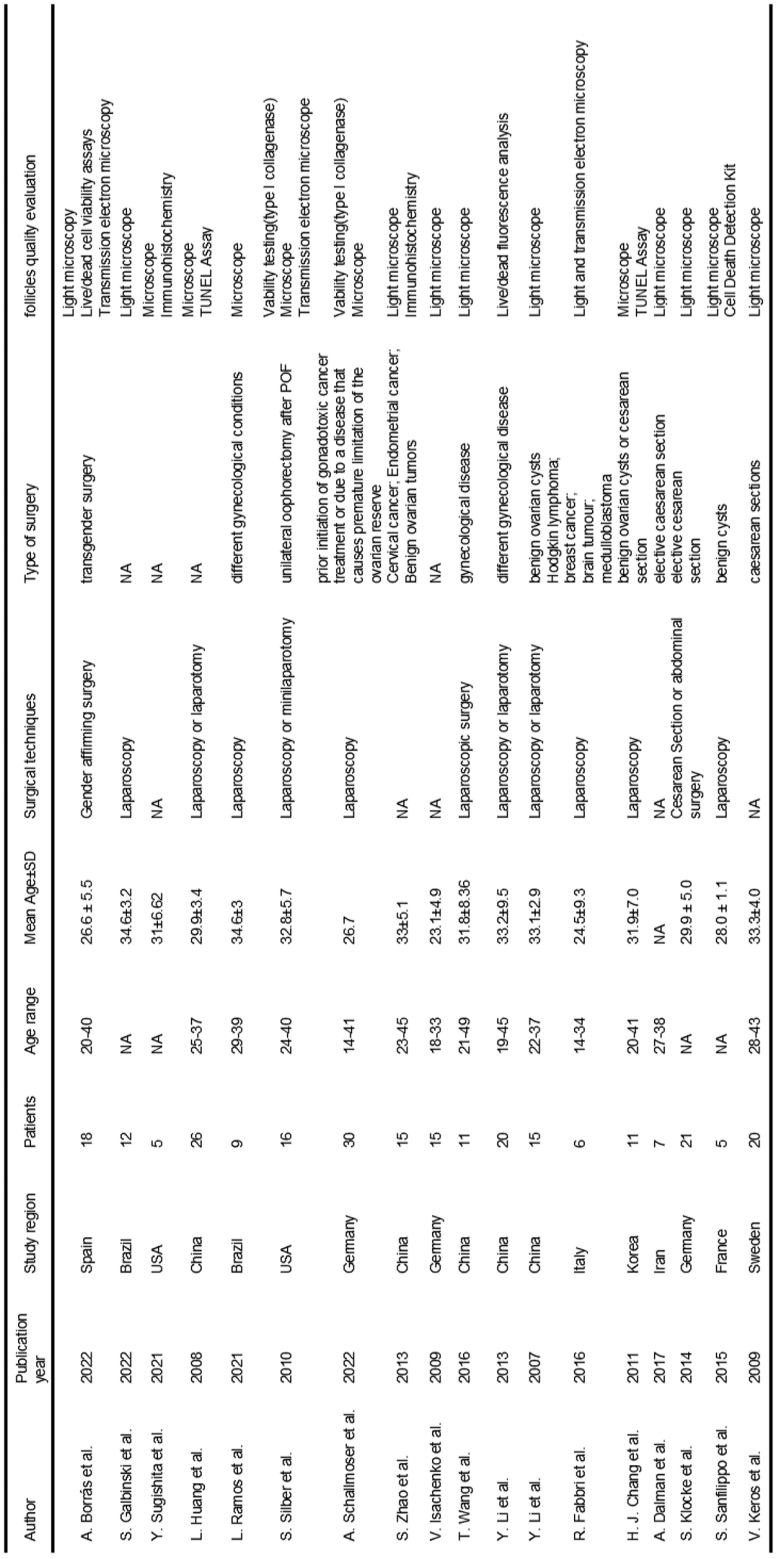
Main characteristics of the selected researches

**Table 2:**
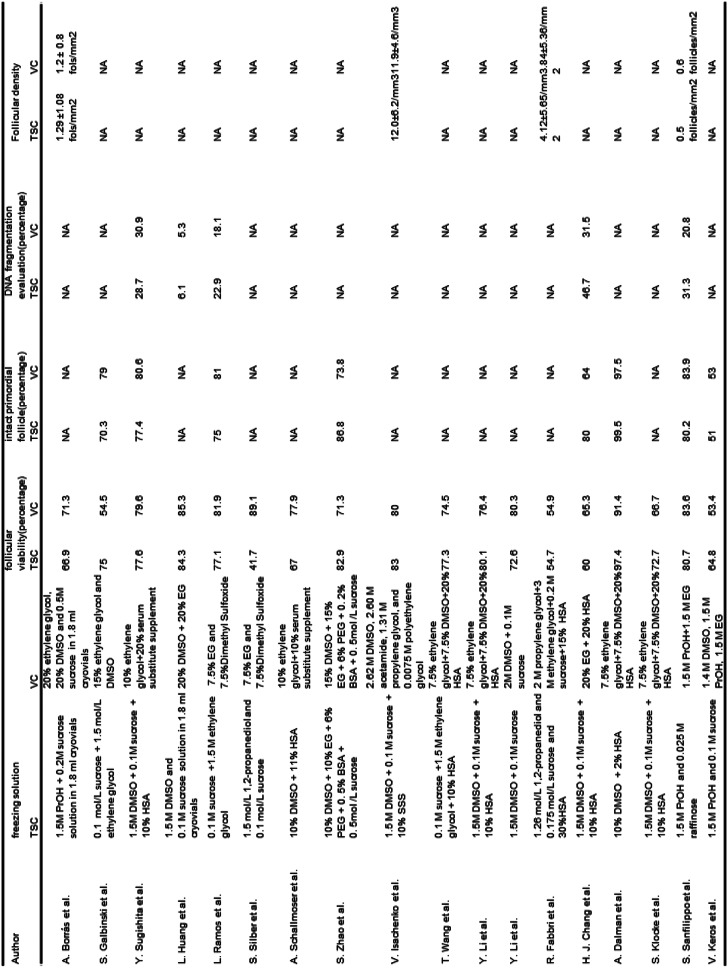
Main method and outcome of the selected researches

### Primary outcome

#### Follicular viability

**Fig. 3:**
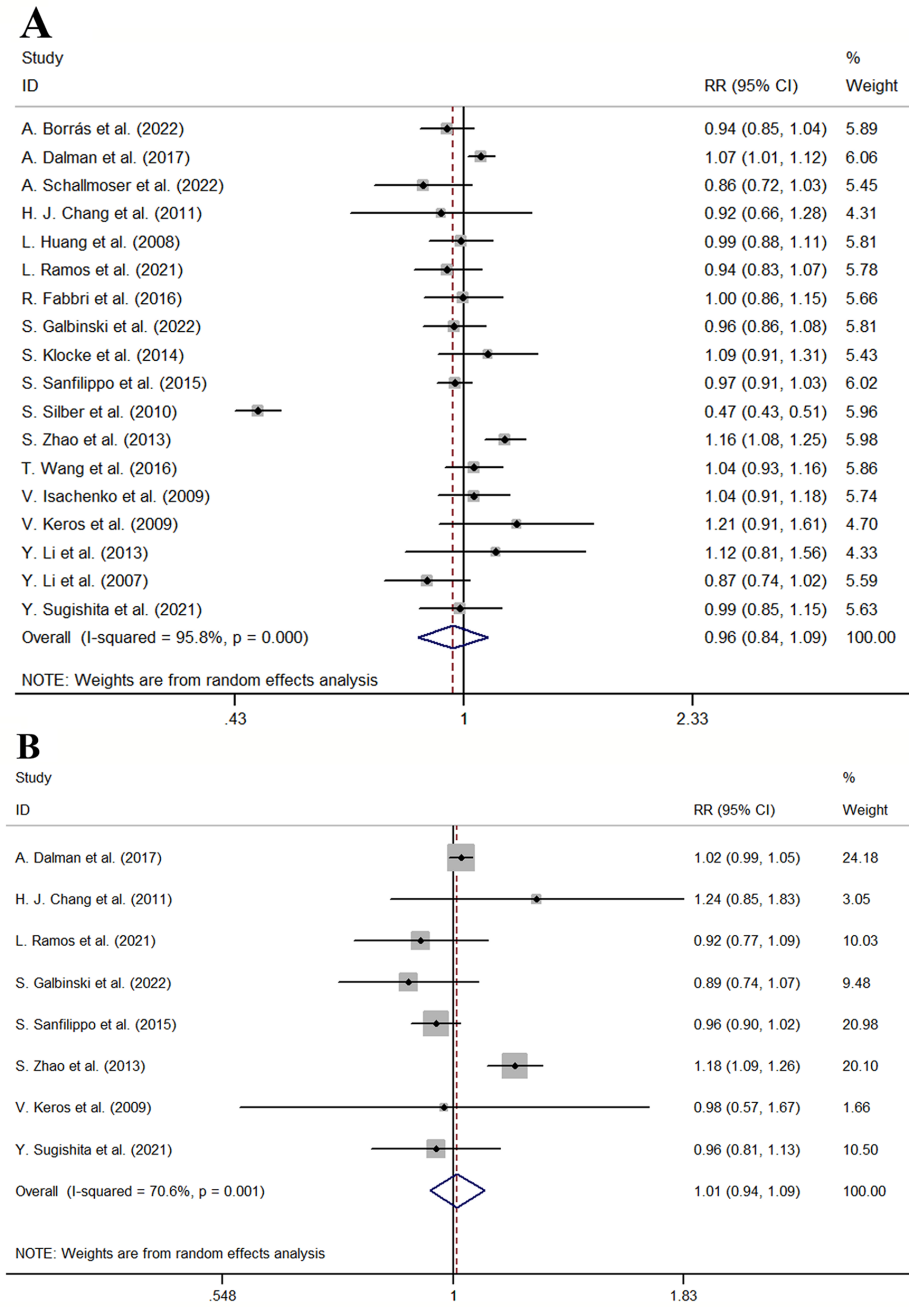
Forest plot for primary outcome (**A**) Forest plot for the follicular viability of traditional slow cryopreservation and vitrification (**B**) Forest plot for the proportion of intact primordial follicles of traditional slow cryopreservation and vitrification

The follicular viability of conventional slow cryopreservation versus vitrification in the 18 publications was analyzed. The pooled results indicated that there was no difference in follicular viability between the two approaches from random effect analysis (RR = 0.96, 95% CI: 0.84–1.09, I^2^ = 95.8%, *P* < 0.001 for heterogeneity, *P* = 0.520 shown in Fig. 3A). S.Silber et al.’s study reported vitrification was associated with significantly higher follicular viability (RR = 0.47, 95% CI: 0.43–0.52) [40]. Other two studies from A. Dalman et al. (RR = 1.07, 95% CI: 1.01–1.12) and S.Zhao et al. (RR = 1.16, 95% CI: 1.08–1.25) reported conventional slow cryopreservation was associated with higher follicular viability [42, 49]. Other studies reported there was no difference in follicular viability between the two approaches.

### The proportion of intact primordial follicles

The proportion of intact primordial follicles of conventional slow cryopreservation versus vitrification in the 8 publications was analyzed. The other 10 publications did not report the data of the proportion of intact primordial follicles. The pooled results indicated that there was no difference in the proportion of intact primordial follicles between the two approaches from random effect analysis (RR = 1.01, 95% CI: 0.94–1.09, I^2^ = 70.6%, *P* = 0.001 for heterogeneity, *P* = 0.778 shown in Fig. 3B). S.Zhao et al.’s study reported conventional slow cryopreservation was associated with higher intact primordial follicles (RR = 1.18, 95% CI: 1.09–1.26) [42]. Other studies reported there was no difference in intact primordial follicles between the two approaches.

### Second outcome

#### The proportion of DNA fragmented follicles

**Fig. 4:**
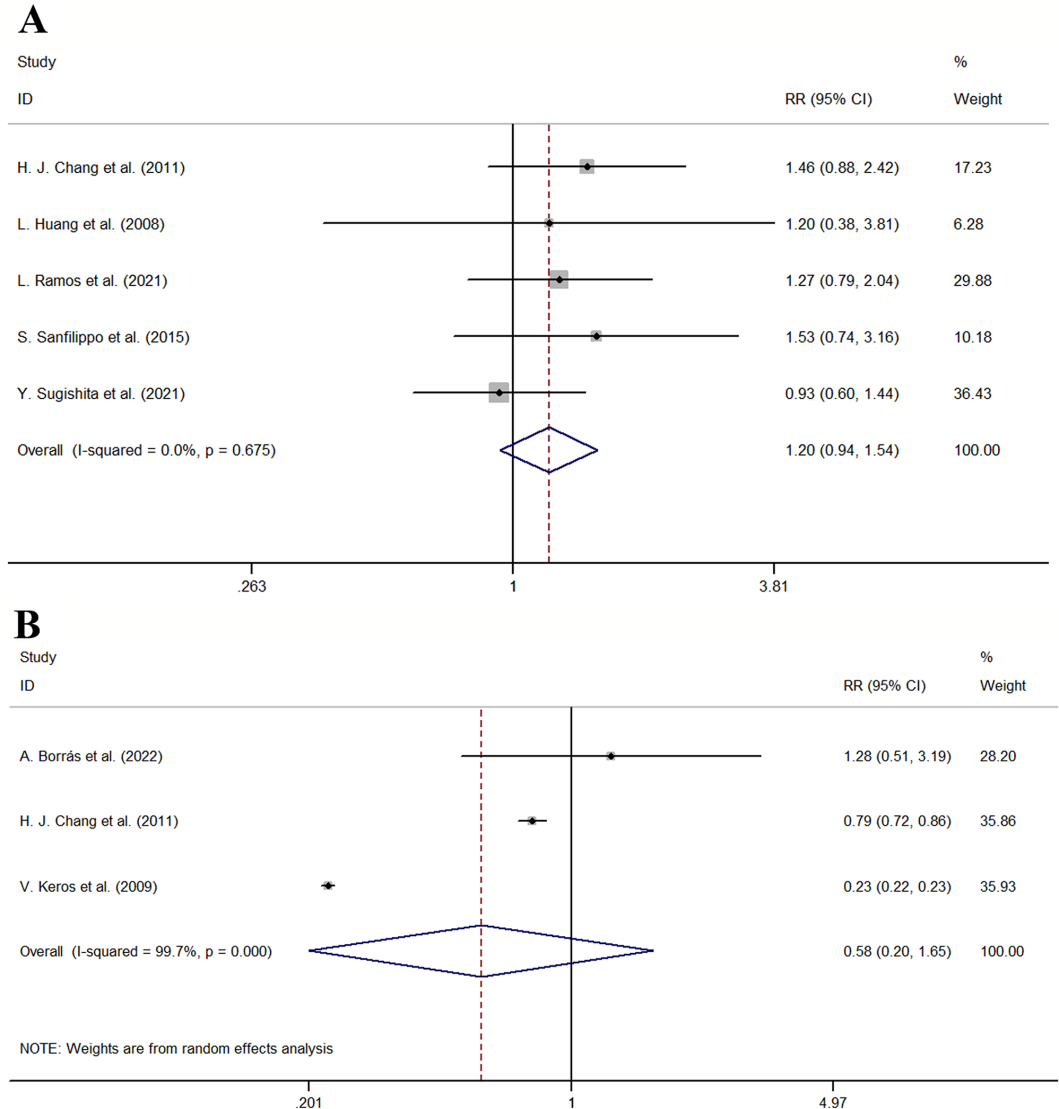
Forest plot for second outcome (**A**) Forest plot for the proportion of DNA fragmented follicles of traditional slow cryopreservation and vitrification (**B**) Forest plot for the proportion of stromal cells of traditional slow cryopreservation and vitrification

The proportion of DNA fragmented follicles of conventional slow cryopreservation versus vitrification in the 5 publications was analyzed. The other 13 publications did not report the data of the proportion of DNA fragmented follicles. The pooled results indicated that there was no difference in the proportion of DNA fragmented follicles between the two approaches (RR = 1.20, 95% CI: 0.94–1.54, I^2^ = 0.0%, *P* = 0.675 for heterogeneity, *P* = 0.151 shown in Fig. 4A).

### The proportion of stromal cells

The proportion of stromal cells of conventional slow cryopreservation versus vitrification in the 3 publications was analyzed. The other 15 publications did not report the data of the proportion of stromal cells. The pooled results indicated that there was no difference in the proportion of stromal cells between the two approaches from random effect analysis (RR = 0.58, 95% CI: 0.20–1.65, I^2^ = 99.7%, *P* < 0.001 for heterogeneity, *P* = 0.303 shown in Fig. 4B). The study from V. Keros et al. (RR = 0.23, 95% CI: 0.22–0.23) and H. J. Chang et al. (RR = 0.79, 95% CI: 0.72–0.86) reported vitrification was associated with significantly higher stromal cells [48, 52]. The other study reported there was no difference in the proportion of stromal cells between the two approaches.

### Risk of bias in included studies

**Fig. 5:**
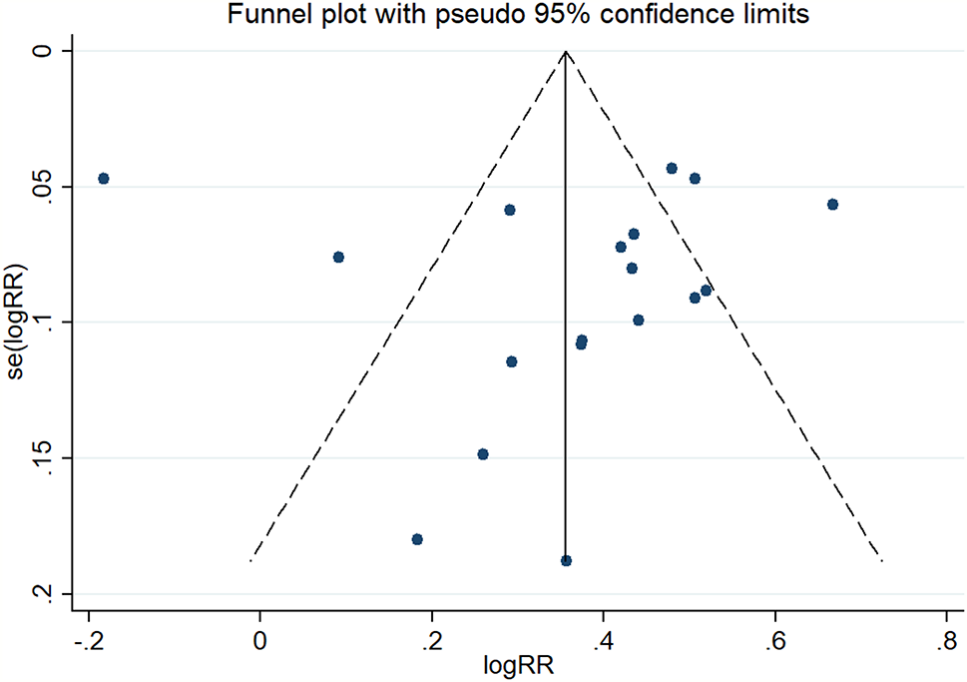
Funnel plot bias of the studies included in this meta-analysis

**Fig. 6:**
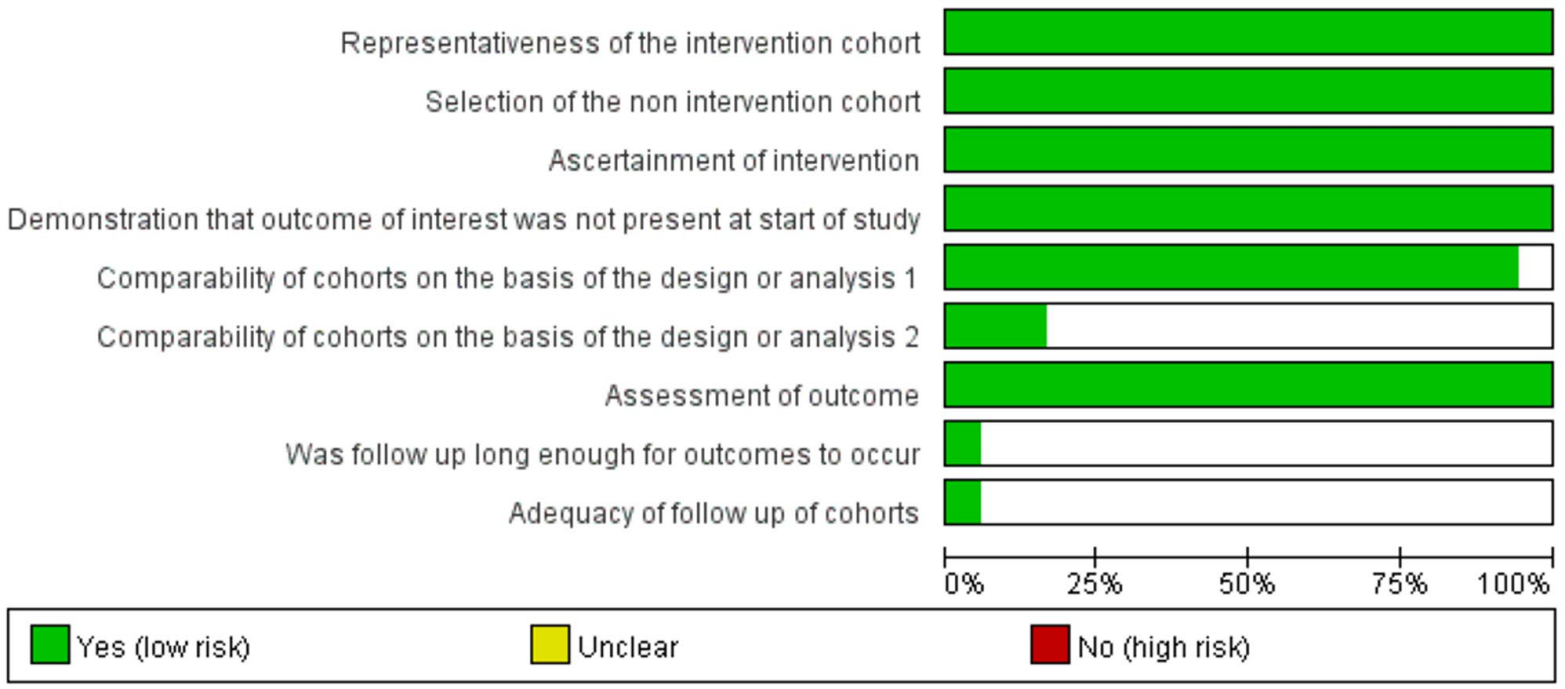
Risk of bias graph presenting authors’ judgments according to Newcastle-Ottawa Scale for evidence-based medicine checklist about each risk of bias item as percentages across all included studies

Funnel plot bias of the studies included in this meta-analysis was notably symmetrical indicating that there was no obvious bias in publication shown in Fig. 5. According to the Newcastle-Ottawa Scale for evidence-based medicine checklist about each risk of bias item as percentages across all included studies, the risk of bias graph was shown in Fig. 6.

## Discussion

The meta-analysis is to compare the quality of ovarian tissue cryopreservation by conventional slow cryopreservation and vitrification. A total of 18 articles met the criteria were included. No more articles were included due to that ovarian tissue cryopreservation is not a routine method for every reproductive center, more centers use oocyte cryopreservation to preserve patients’ fertility [53, 54]. First, due to complex ethical methods, ovarian tissue cryopreservation has not yet become a routine method recognized by the public. Second, oocyte cryopreservation can meet the basic requirements of patients for preserving fertility. The cryopreservation of ovarian tissue aims to better preserve endocrine function, allowing follicles to mature and develop more effectively, thereby more accurately maintaining the patient’s fertility [55–58]. In recent years, with advancements in science and technology and increased awareness, the number of ovarian tissue cryopreservation has increased gradually [27, 52, 59, 60]. There is also controversy about the method of ovarian tissue cryopreservation [61]. Conventional slow cryopreservation can minimize the formation of intracellular ice crystals during the freezing process through gradient cooling. To ensure cell viability, this method has been widely used since the development of ovarian cryopreservation and has achieved good results. The first ovarian transplant birth was the application of conventional slow cryopreservation [62]. However, conventional slow cryopreservation takes a long time and leads to cells death, especially increased [63]. Recently, vitrification has been gradually popular. The vitrification can freeze tissues more quickly though speeding up the freezing by increasing the concentration of cryoprotectant. However, vitrification will lead to the loss of cytoskeletal elements and is more likely to lead to cellular ischemia [64, 65]. As whether the newer freezing method can replace the conventional slow cryopreservation method has become a topic of significant consideration.

### The primary outcomes

#### Follicular viability

As we all know, in ovarian cryopreservation, the most important evaluation criterion is follicular viability [28, 66, 67]. Follicular viability is the basis for determining the patient’s subsequent fertility and is also an important indicator for evaluating ovarian cryopreservation methods. Comparing follicular viability between the two cryopreservation methods, we find that S. Silber et al.‘s study reported significantly higher follicular viability with vitrification, with viability rates of 41.7% for slow freeze-cryopreserved tissue and 89.1% for vitrified tissue [40]. This is quite different from other studies. Without subjective bias, we analyze the reasons for the difference of follicular viability in conventional slow cryopreservation and vitrification. First, in this article, the freezing solution of conventional slow cryopreservation did not contain human serum albumin (HSA) a cryoprotectant that helps protect cells by reducing cellular ice crystal formation [68]. The article was published in 2010, and followed the ovarian transplants for up to 5 years, so the method may be used in 2005 or before. The HSA may not be used in those years as cryoprotectant. Recently, HSA has been widely used for ovarian tissue cryopreservation. In addition to reducing the formation of intracellular ice crystals, it can also maintain cell osmotic pressure and enhance cellular antioxidant capacity [69]. Although it is not an essential component of cryoprotectants, with it can reduce cell damage. Second, the follicles quality evaluation method of this article is different from other articles. Most other articles use microscope counting to judge. This article uses propidium iodide (PI) staining and flow cytometry for detection and assessment. Microscopic counting may not effectively observe subtle changes in cell apoptosis, as early-stage apoptotic cells are harder to identify. However, PI staining can accurately identify cell status. Additionally, transmission electron microscopy is used to observe the cells in this study. The result is that most of the stroma cells in the slow freeze–cryopreserved specimen was lysed and their nuclei compressed between dense bundles of extracellular fibers. Stroma cells are very important in the follicle maturation process, so it may be that the damage to stromal cells caused by conventional slow cryopreservation further led to the damage to follicle cells [70, 71].Third, the freezing cryoprotectant of vitrification is 7.5% ethylene glycol (EG) and 7.5% DMSO for 25 min followed by a second equilibration in 20% EG and 20% DMSO for 15 min. The first concentration is lower than other vitrification methods. Additionally, synthetic serum substitute (SSS) was added as a protective agent, which may contribute to better follicular viability. However, A. Dalman et al. and S.Zhao et al. reported conventional slow cryopreservation was associated with higher follicular viability. A. Dalman et al. reported the follicular viability of conventional slow cryopreservation was 97.4% and vitrification was 91.4% [49]. The follicular viability in this study is higher than in other studies. This study uses microscope counting for detection and assessment. There may be some subjective bias. At the same time, vitrification was correlated with more free radicals, and reactive oxygen species (ROS) [72]. Oxidative stress occurs when more ROS produced, which will result in damage to cellular components such as DNA, proteins, and lipids and cause cell damage [73, 74]. Adding catalase to the cryoprotectant may reduce the production of ROS and protect freezing cells [75]. S.Zhao et al. reported the follicular viability of conventional slow cryopreservation was 82.9% and vitrification was 71.3% [42]. The main reason for this difference is that this study used large pieces of human ovarian tissues that is 15 mm × 15 mm × 2 mm which is larger than other studies. The larger ovarian tissue cryopreservation may reduce follicles loss and support follicles growth [76, 77]. The higher density of the vitrification cryoprotectant prevents it from quickly penetrating large tissues, resulting in uneven vitrification of the cellular water throughout the tissue. This may be the reason for conventional slow cryopreservation was associated with higher follicular viability.

#### The proportion of intact primordial follicles

Primordial follicles are the earliest stage of ovarian follicles, which account for more than 90% of the population of follicles [78, 79]. During ovarian tissue cryopreservation, a higher proportion of intact primordial follicles indicates greater fertility potential [43, 80]. Comparing the proportion of intact primordial follicles between the two cryopreservation methods, S.Zhao et al. reported the proportion of intact primordial follicles of conventional slow cryopreservation was 86.8% and vitrification was 73.8% [42]. As previously mentioned, this study used large pieces of human ovarian tissues, which may lead to differing results between the two methods. Larger tissue samples may benefit from conventional slow cryopreservation not vitrification, which may not penetrate large tissues quickly enough, potentially leading to the formation of intracellular ice crystals that can damage cells. More studies about this supposed need to be verified. Additionally, while the morphology of frozen primordial follicles may appear normal, their subsequent development, fertilization, and the ability to achieve pregnancy and live birth can be influenced by various factors [54, 81]. Therefore, fertility outcomes cannot be solely determined by normal morphology; it represents just the initial step in assessing the efficiency of cryopreservation.

### Secondary outcomes

#### The proportion of DNA fragmented follicles

According to this meta-analysis, there was no difference in the proportion of DNA fragmented follicles between the two approaches. Both methods can lead to cell apoptosis, mainly manifested as DNA damage. This is mainly attributed to the inevitable formation of intracellular ice crystals during the freezing process, despite efforts by cryoprotectants to minimize their formation. The formation of intracellular ice crystals can cause mechanical damage, which can further lead to rupture of cell membranes and other cellular structures. Moreover, it can cause structural damage by causing damage and breakage of DNA strands due to reduced space for cell expansion [82, 83]. In addition, cryoprotectants containing that may cause chemical damage and directly damage DNA [84]. Therefore, reducing DNA damage during the freezing process should also be considered in future freezing methods.

#### The proportion of stromal cells

Stromal cells are an essential part of ovarian tissue providing structural support for follicles and contributing to the production of extracellular matrix components and hormones. They provide the necessary microenvironment for the growth and development of oocytes and play an important role in the development and maturation of follicles [85, 86]. Research shows that the weakened antioxidant capacity of stromal cells is closely related to ovarian aging [87]. More attention should be paid to the survival status of stromal cells in ovarian tissue cryopreservation. The study of H. J. Chang et al. showed that the proportion of stromal cells of conventional slow cryopreservation was 47.8% and vitrification was 60.7%. Vitrification may reduce damage to stromal cells, possibly because stromal cells are relatively small. During vitrification, they can quickly reach the vitrified state, reducing the formation of intracellular ice crystals, and thereby reducing cell damage [69]. However, it’s important to note that the vitrification method is not standardized, and further studies using consistent vitrification protocols are needed for comprehensive evaluation.

### Limitations

This meta-analysis reviewed the comparison of ovarian tissue cryopreservation by conventional slow cryopreservation and vitrification till January 2024. Our results indicate comparable outcomes for ovarian tissue cryopreservation between the two approaches. The main limitation of our study is the significant variation in the methods of conventional slow cryopreservation and vitrification. This variability arises from differences in the composition and ratio of cryoprotectants used. They mainly contain such as EG, DMSO, PrOH, etc. The difference between the different cryoprotectants is not particularly obvious. There is currently no unified regulation. Studies by Meryman indicated that osmotic effects may be the major factors in slow-freezing cell injury [88]. The same group demonstrated that adding neutral solutes as cryoprotective agents (CPAs) can reduce osmotically driven cell injury during ice solidification due to colligative effects [89]. There are two main categories of cryoprotective agents [90, 91]. The first is penetrating cryoprotective agents. DMSO is one of the most effective and widely used cryoprotective agents which is a small, amphipathic molecule that can easily penetrate cell membranes and distribute throughout both intracellular and extracellular environments [92]. During the freezing process, DMSO creates an osmotic gradient that helps dehydrate cells, reducing the amount of free water inside them and thereby decreasing the chances of intracellular ice formation. By controlling the osmotic balance, it also helps prevent osmotic shock during freezing and thawing, which can lead to cell lysis or severe deformation. The concentration of DMSO used in cryopreservation typically ranges from 5 to 10% [93–95]. EG is another effective cryoprotective agent that has a similar function to DMSO. The main difference is that EG is a small, water-soluble molecule. The concentration of ethylene glycol used in cryopreservation protocols typically ranges from 10 to 20% [96, 97]. In many studies, a mixture of DMSO and EG is reported to be more effective as the cryoprotectant [93, 98–100]. The other cryoprotective agents are non-penetrating cryoprotective agents with sucrose being the primary one. The combination of different cryoprotectants aims to enhance osmotic pressure regulation inside and outside cells, prevent intracellular ice crystal formation, and stabilize cell membranes and proteins. Different researchers utilize varying combinations of cryoprotectants based on their individual research experiences [93, 94, 101, 102]. Moreover, follicular viability, the proportion of intact primordial follicles, the proportion of DNA fragmented in follicles, and the proportion of stromal cells are essential criteria for evaluating ovarian tissue cryopreservation. The clinical pregnancy rate is also very important in ovarian tissue cryopreservation and is the most crucial metric for evaluating female reproductive function. However, under current clinical conditions, experience is relatively limited. Beyond evaluating ovarian cryopreservation technology, the success of ovarian tissue cryopreservation also depends on transplantation surgery techniques, the method of conception, and the physical condition of the female patient, including factors such as pelvic radiation and prior chemotherapy. And until now, the ovarian tissue cryopreservation is considered an experimental procedure. More studies are needed.

## Conclusion

In conclusion, this meta-analysis indicated that conventional slow cryopreservation and vitrification appear to provide comparable outcomes of follicular viability, the proportion of intact primordial follicles, the proportion of DNA fragmented follicles and the proportion of stromal cells. However, included studies varied in the use of cryopreservation protocols. Therefore, further studies are needed to determine the optimal method for cryopreservation human ovarian tissue. While there is no significant difference between traditional cryopreservation and vitrification regarding ovarian tissue anatomy, each method has its own advantages and is suitable for different clinical scenarios. The advantages of traditional cryopreservation include its mature technology and relatively standardized procedures. On the other hand, vitrification offers benefits such as fast freezing speed, simplicity of operation, and high flexibility. And until now, the ovarian tissue cryopreservation is considered an experimental procedure. The choice of method can be tailored to meet specific clinical needs.

## Data Availability

No datasets were generated or analysed during the current study.

## Abbreviations

DMSO: Dimethyl sulfoxide
PrOH: Glycerol, propylene glycol
EG: Ethylene glycol
HAS: Human serum albumin
SSS: Synthetic serum substitute
ROS: Reactive oxygen species
